# The experience of transition preparation for children and adolescents with mental disorders in China: a qualitative study

**DOI:** 10.3389/fpsyg.2025.1638296

**Published:** 2025-09-01

**Authors:** Yingying Miao, Juan Li, Jihong Wang, Xiaohuan Zhou, Xin Li, Yi Zhang, Hui Xu

**Affiliations:** ^1^Nursing School, Pingdingshan University, Pingdingshan, China; ^2^Nursing and Health School, Zhengzhou University, Zhengzhou, China

**Keywords:** primary care, transition, adolescents, mental health, qualitative study

## Abstract

**Background:**

Adolescence represents a critical transitional phase from pediatric to adult healthcare, during which young individuals with mental disorders encounter unique challenges; however, research on their transition experiences, particularly in non-English-speaking contexts such as China, remains limited.

**Objectives:**

This study aimed to explore the transition preparation experiences of adolescents with mental disorders in China as they move from pediatric to adult healthcare.

**Methods:**

Qualitative semi-structured interviews were conducted with 15 children and adolescents diagnosed with mental disorders in China, and the data were analyzed using interpretive phenomenological analysis.

**Findings:**

The study identified five primary themes related to the transition experiences of children and adolescents with mental disorders: (1) a discrepancy between transitional awareness and action; (2) gaps in transitional care throughout the healthcare transition process; (3) multidimensional needs within transitional care; (4) a contradiction between desire for self-management of illness and limited capacity; and (5) the dual attributes of family support, encompassing both enabling and constraining aspects.

**Conclusion:**

The study underscores the concerning transition readiness of adolescents with psychiatric disorders in China, marked by complex challenges and diverse needs, while highlighting the dual role of family support as both a motivating and obstructive force.

## Background

Childhood and adolescent mental disorders (CAMDs) are chronic conditions of uncertain etiology that arise during childhood and adolescence and are characterized by profound physiological, psychological, and social changes, such as immaturity, emotional instability, and heightened vulnerability to environmental influences (Piao et al., 2022). According to 2021 WHO statistics, mental disorders affect approximately 13% of individuals aged 10–19 worldwide, representing one in seven people within this age group (Bellomo et al., 2023). An epidemiological survey in China found that 17.5% of children and adolescents aged 6–16 had mental disorders, indicating an increasing trend (Li et al., 2022). These disorders substantially affect physical, social, and cognitive development during adolescence and may have lasting consequences across the lifespan.

Adolescence is a pivotal developmental stage marked by significant physical, psychological, and social changes. For adolescents with mental disorders, this period presents added complexity, as they must cope with both typical developmental tasks and the transition from pediatric to adult healthcare systems (Ogundele and Morton, 2022). Addressing their unique needs is essential for effective support. This transition often involves treatment adjustments, changes in providers, and the development of self-management skills (Paton et al., 2022). While pediatricians or child psychiatrists typically oversee care during early adolescence, adult mental health specialists assume responsibility as individuals mature. Ensuring continuity of care requires effective communication between providers and the timely transfer of medical records (Nadarajah et al., 2021). Successfully navigating this process is crucial for long-term health outcomes and quality of life.

Recent literature highlights the growing importance of transitional care in pediatric healthcare (Richter Sundberg et al., 2021). In mental health, transition refers to the structured shift from child and adolescent to adult mental health services (Göbel et al., 2022). Transition readiness entails a planned approach that addresses the specific needs of adolescents with chronic mental or physical conditions as they move to adult-centered care (Leeb et al., 2020). Proper preparation is crucial to prevent treatment disruptions, reduce disengagement, and promote adherence, ultimately improving outcomes and quality of life (Bellomo et al., 2023). Nurses play a key role in this process, with a professional duty to deliver holistic, developmentally appropriate care in line with pediatric standards (van Driessche et al., 2021). A well-managed transition supports continuity, enhances treatment effectiveness, and contributes to positive long-term health outcomes.

Research on transition readiness among children and adolescents with mental illness has predominantly been conducted in Western countries. Several studies have examined the transitional experiences of this population in nations such as the United States (Okumura et al., 2022) and Canada (Toulany et al., 2024). Findings indicate that the preparation for healthcare transitions among adolescents with chronic conditions remains inadequate. For instance, approximately 64% of adolescent patients reported a lack of clarity regarding their healthcare needs during the transition from pediatric to adult services. Furthermore, as many as 43% of adolescents who experienced disruptions in mental healthcare during the shift to adult mental health services did not receive continuous care throughout the transition process (Syverson et al., 2016). Additional research suggests (Every-Palmer et al., 2022) that a substantial number of young people require a transition from child and adolescent mental health services to adult services; however, this process is often fragmented. Many adolescents lack a comprehensive understanding of the transition and frequently report negative experiences and limited awareness of the factors that contribute to a successful transition. Therefore, it is essential to explore the challenges and barriers associated with transitional care in greater depth and to identify key influencing factors. This understanding is critical for developing effective, evidence-based practices that support optimal transitions in mental healthcare.

Despite growing interest in healthcare transitions, limited research has explored the experiences of children and adolescents in China. Existing studies have primarily focused on specific groups, such as adolescents with epilepsy (Cui et al., 2020) or premature infants (Ma et al., 2021). For example, research indicates that young epilepsy patients in China often lack adequate preparation for transition and need greater involvement in the process (Cui et al., 2020). However, there is a notable absence of studies examining the transition experiences of youth with mental illnesses. This gap hinders the identification of their specific challenges and impedes the development of targeted interventions and policies to support smoother transitions and to improve long-term mental health outcomes.

In addition, it is crucial to develop intervention programs for transitional care. Currently, there is a lack of specific intervention programs in China to provide information support for such initiatives. Although medical staff are aware of the importance of transitional care for adolescent patients, most medical staff lack relevant policies or structured transitional guidance programs, which may lead to insufficient provision of transitional care (Yang et al., 2025). Investigating the transition of children and adolescents with chronic diseases in primary care will be a focus of future research. Given the significant cultural differences between China and the West, it is crucial to conduct in-depth research on the transitional experiences, subjective cognitions, and emotional responses of children and adolescents with mental disorders in China. By fully understanding their experiences, we can develop more targeted and culturally sensitive nursing interventions to meet their specific needs and help them transition to adulthood more smoothly. In addition, exploring transitional experiences will contribute to the advancement of mental healthcare practices in China and provide valuable reference for future research on transitional policies for children and adolescents with mental disorders.

Based on these considerations, this qualitative study aims to explore the transitional preparation experiences of children and adolescents with mental disorders in the People’s Republic of China. Through in-depth interviews with patients with mental disorders, we seek to gain a deeper understanding of their perspectives, challenges, and needs during the transition process. Through this exploration, we aim to enrich the existing literature on healthcare transitions for adolescents with mental disorders, especially in the context of the Chinese healthcare system. Understanding their transition experiences is essential for designing personalized interventions and support programs that meet their unique needs.

## Methods

Interpretative phenomenological analysis (IPA) (Ugiagbe et al., 2025) guided the study’s design, data collection, and analysis. Rooted in psychology, IPA is a qualitative approach ideal for examining how individuals interpret significant life experiences. It typically involves small sample sizes (e.g., 4–15participants) to enable detailed, idiographic analysis. Semi-structured, one-on-one interviews are central to IPA, offering rich, first-person insights into participants’ lived experiences. Accordingly, IPA was deemed appropriate for this study in order to investigate the transition experiences of children and adolescents with mental disorders. Ethical approval for this research was granted by the Research Ethics Committee of Pingdingshan University (PSDU2023-018).

### Study design

The interviews were conducted by one of the study authors (Miao). Prior to each interview, the researcher established rapport with the participants to foster a comfortable and open environment. Participants were informed that the researcher was interested in understanding their current care experiences with the aim of improving patient care in the future. Semi-structured interviews were employed to assess the readiness for healthcare transition among children and adolescents with mental disorders. This qualitative approach enabled a detailed exploration of participants’ lived experiences, offering valuable insights into their perceptions, challenges, and needs during the transition process.

### Participant recruitment

Fifteen patients from a psychiatric ward in Zhengzhou City, China, participated in this study. The participants, who represented a diverse range of psychiatric diagnoses, were predominantly of Asian ethnicity. A total of 16 individuals were initially approached and interviewed, with 15 ultimately included in the final analysis. Participants were selected using purposive sampling from a single hospital. Informed consent was obtained from all participants prior to data collection. They were informed of their right to withdraw from the interview at any time without consequence and were assured that their data would be deleted if they chose to do so.

The eligibility criteria included the following: (1) a confirmed diagnosis of a mental disorder according to the International Classification of Diseases, 10th Edition (ICD-10), verified through interviews by two psychiatrists with the title of attending physician or higher. Eligible diagnoses included anxiety disorder, emotional disorder, depression, obsessive-compulsive disorder, and mood disorder, among others; (2) age between 12 and 18 years; (3) the ability to read and comprehend written language; (4) provision of informed consent by both the participant and their legal guardian; and (5) completion of at least a primary school level of education.

The exclusion criteria were as follows: (1) the presence of a serious physical illness; (2) a history of psychoactive substance-induced seizures, substance abuse, bipolar disorder, manic episodes, or unstable psychiatric conditions; and (3) comorbidities such as severe personality disorders, significant cognitive impairments, or other psychiatric conditions that could complicate the primary diagnosis or interfere with the transitional care process. Participants’ demographics and diagnoses are presented in Table 1.

**Table 1:** Demographic characteristics and diagnostic information of participants (*n* = 15).

Characteristics	Adolescents
Gender	
Female	9
Male	6
Education	
Middle school	9
High school	6
Junior college	1
Disease diagnosis	
Anxiety disorder	5
Emotional disorder	3
Depression	5
Obsessive-compulsive disorder	1
Mood disorder	1
Duration (months)	
<3	6
3–6	3
6 ~ 12	2
>12	4
Grade (currently enrolled)	
1	11
2	3
≥3	1
Only Child	
Yes	5
No	10

### Data collection

Interviews were conducted from April to June 2023 in a quiet, private room within the psychiatric ward to ensure participant comfort and confidentiality, fostering an environment conducive to open and uninterrupted expression. Each session was conducted one-on-one between the researcher and the participant to uphold strict confidentiality. The interviewer used follow-up prompts, as required, to encourage elaboration and clarify responses. All interviews were audio-recorded using a voice memo application, and supplementary field notes were taken to capture non-verbal cues and contextual details. Data collection was continued until thematic saturation was achieved, defined as the point at which no new concepts or themes emerged. Data saturation was achieved after 15 interviews. To enhance credibility, the researcher maintained a reflective journal to record personal insights, track analytic decisions, and acknowledge potential biases. Additionally, participants engaged in member checking by reviewing and commenting on the initial themes derived from their interviews, thereby helping to validate the accuracy and authenticity of the findings.

The interview outline is as follows: (1) What do you know about the process of transitioning from healthcare for children to healthcare for adults? (2) What did you do to prepare for the transition to adult healthcare? (3) What factors influenced your preparation for the disease transition (positive/negative factors)? (4) What challenges or difficulties did you encounter during the transition? (5) How do you plan to address these challenges/difficulties? (6) What problems do you see with transitional care in the current healthcare system? What are your best suggestions? And (7) What assistance would you like to receive during the transition? What are your expectations?

### Data analysis

Interpretative phenomenological analysis was employed to analyze the Chinese verbatim transcripts. The researcher systematically coded each transcript, developing a hierarchical structure of emergent themes relevant to the research question, and presented them in a tabular format for each individual transcript (Ugiagbe et al., 2025). An iterative analytical process enabled the development of a comprehensive thematic framework reflecting participants’ lived experiences. MAXQDA software was used for data management, indexing, and retrieval. Representative anonymized quotes are presented to illustrate each theme. To ensure credibility and trustworthiness, a rigorous cross-checking procedure was employed: two Chinese transcripts were independently coded by the first author and reviewed by the third author. Example excerpts were then translated into English by the first author and verified for linguistic and conceptual accuracy by a bilingual researcher. Additionally, two initial transcripts were independently coded by two researchers to assess consistency and inter-rater reliability. Any discrepancies in coding were discussed and resolved through consensus. However, when disagreements persisted, a full research team meeting was convened to deliberate on the codes and reach an agreement, ensuring analytical rigor and transparency.

### Findings

**Figure 1:**
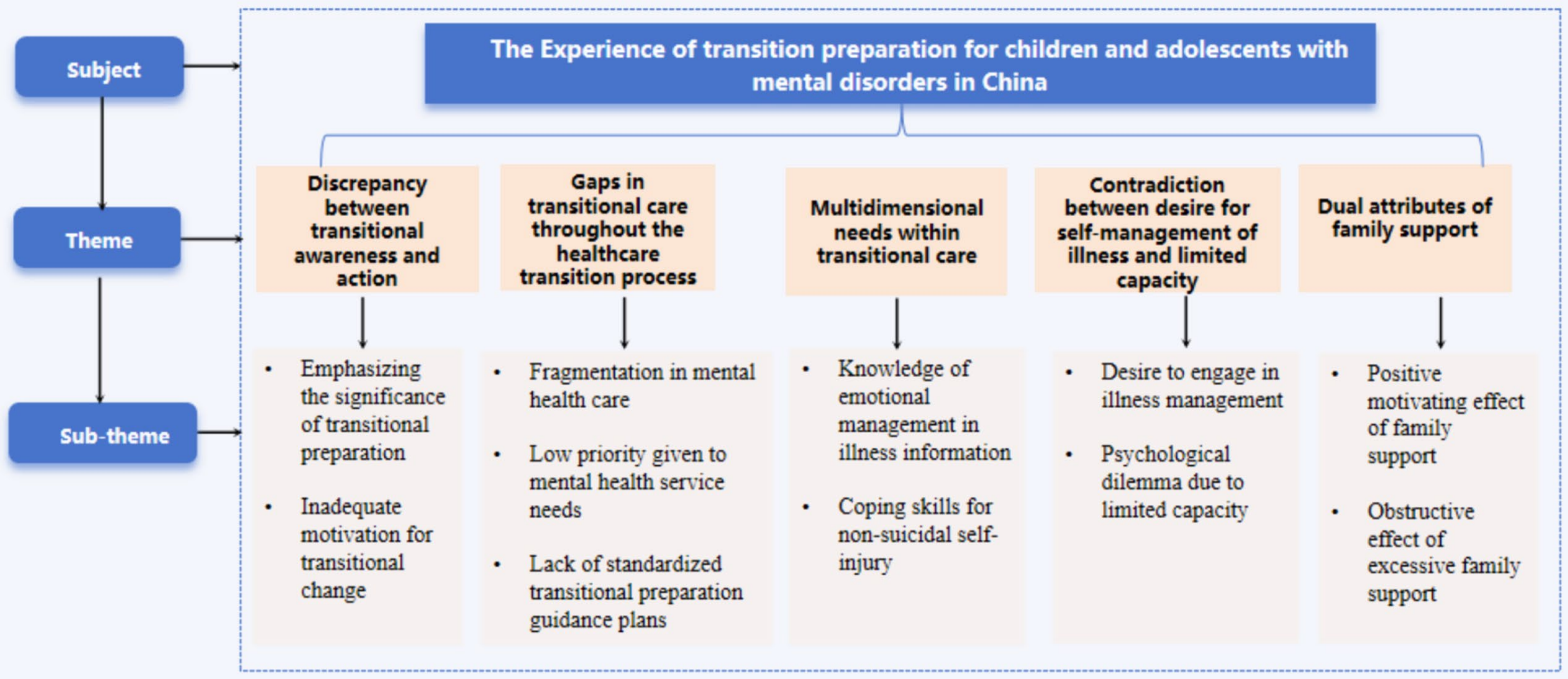
Themes and subthemes identified in the transition experiences of adolescents with mental disorders.

A total of 15 participants ranging in age from 12 to 18 years, with a mean age of 15 years, were included in this study. Detailed demographic and clinical characteristics of the participants are presented in Table 1. The semi-structured, audio-recorded interviews lasted between 25 and 40 min each. Through IPA, 5 overarching themes and 11 corresponding subthemes were identified (Figure 1). The following section presents the key themes and subthemes, along with a summary of the interview findings. Direct quotations from participants were used to illustrate the themes, with identifiers (N1–N15) indicating the specific interview from which each quotation was drawn.

### Discrepancy between transitional awareness and action

Two key subthemes emerged under the overarching theme of discrepancy between transitional awareness and action: (i) emphasizing the significance of transitional preparation and (ii) inadequate motivation for transitional change. The subthemes reveal a key contradiction: although participants acknowledged the importance of preparing for the transition to adult healthcare, many lacked the motivation or confidence to engage actively. This disconnect suggests that, while adolescents with mental health disorders are cognitively aware of the need for transition readiness, emotional and psychological barriers may hinder practical action.

#### Emphasizing the significance of transitional preparation

Adolescents with mental disorders recognized the transition to adult care as a critical period requiring deliberate preparation. Their awareness reflects a cognitive understanding of the challenges involved and highlights the need to acquire the knowledge and skills necessary to navigate this shift effectively.

*N3: “I understand that I’m going to be an adult, and this process is more critical. I can go to the adult clinic in the future. I’m not a child anymore.*

*N5: “What I understand is that the way I used to see a doctor in pediatrics may no longer be suitable for me, and I need to adapt to new changes.”*

#### Inadequate motivation for transitional change

Patients were not adequately prepared for the transition period. The current preparation mainly focuses on regular medication, maintaining a positive mindset, and regular follow-ups. However, detailed, comprehensive, and in-depth planning for the transition period is lacking.

*N4: “I am not ready for discharge because I do not know what will happen after discharge.”*

*N9: “Preparation … After I was discharged from the hospital, I was just taking my medication regularly … Regularly come to the hospital for examination, I was just trying to stabilize my emotions as much as I could, does that count as?”*

### Gaps in transitional care throughout the healthcare transition process

The following subordinate themes were identified: (i) fragmentation in mental healthcare, (ii) low priority given to mental health service needs, and (iii) lack of standardized transitional preparation guidance. Participants reported that mental health services for children and adults are often distributed across separate institutions, leading to fragmented care. This structural divide results in insufficient information sharing and poor coordination, creating significant barriers during the transition process. Furthermore, mental health issues are frequently neglected or marginalized within broader societal and healthcare contexts, with mental health service needs often receiving inadequate attention and resource allocation. Consequently, patients may not receive sufficient support during this critical transitional period. Notably, many participants expressed a lack of access to clear and standardized guidance on how to prepare for the transition. This absence of structured planning further complicated their ability to navigate the shift from pediatric to adult mental health services.

#### Fragmentation in mental healthcare

Children and adolescents with mental disorders require continuity between pediatric and adult healthcare services. However, in practice, this continuity is often disrupted. While patients typically receive regular psychotherapy and psychological care during hospitalization, these services are frequently interrupted upon discharge. Professional psychological support becomes inconsistent, and the healthcare systems serving pediatric and adult populations are often distinctly separated, lacking effective coordination and integration. This structural disconnection hinders the provision of seamless care, potentially compromising the mental health outcomes of transitioning youth.

*N4: “In the hospital, there was a weekly group psychotherapy activity, I attended every session. But once I was discharged, I felt abandoned. No one seemed to care about me anymore. The illness does not just go away because you leave the hospital.”*

*N8: “The community staff would come for home visits, but only to collect information, like my recent emotional state and medication use, and record it. They did not provide any psychological services.”*

#### Low priority given to mental health service needs

During the interviews, adolescent patients with mental disorders frequently expressed that their mental health needs were not given adequate priority during the transition to adult medical care, in contrast to the attention typically afforded to physical health conditions. This finding suggests that mental health concerns may be marginalized or overlooked within the broader framework of transitional healthcare. Many participants reported a lack of sufficient psychological support and continuity of mental health services following discharge. These findings underscore the critical need to recognize and treat mental health as an essential component of overall health, particularly during vulnerable periods, such as the transition from pediatric to adult healthcare.

*N5 shared, “It’s not like there’s something physically wrong with me. My parents just think I’m daydreaming, they do not see mental illness as a real disease.”*

*N6 expressed, “No one wants to listen to my concerns. The doctors just prescribe medication, but emotionally, I feel terrible. Who really cares about how I’m feeling inside?”*

#### Lack of standardized transitional preparation guidance plans

Patients frequently reported feelings of confusion and helplessness during the transition process. Many expressed uncertainty regarding the next phase of their treatment and lacked clarity about how to navigate the shift to adult healthcare in a systematic and standardized manner. Participants consistently emphasized the need for professional, detailed guidance plans to support transition preparation. Such structured plans would help them understand the necessary steps and allow for comprehensive self-preparation. These findings highlight the critical role of formalized guidance and support systems in ensuring effective and smooth healthcare transitions for adolescents with mental disorders.

*N4: “It would be nice if someone could guide me during this period. What can I do to prepare myself and do what I can.”*

*N15: “I had guidance from doctors and nurses in the hospital, after I was discharged from the hospital, I did not know what to do myself, I was more confused, if someone had told me what to plan for next, what to do, I should have been better faster.”*

### Multidimensional needs within transitional care

The following subordinate themes were identified: (i) knowledge of disease information for emotional management and (ii) coping skills for negative emotions. These findings underscore the multifaceted nature of patient needs, particularly in the area of emotional regulation. Adolescents expressed a desire to better understand their mental health conditions as a foundation for managing emotional distress. Additionally, they emphasized the importance of acquiring practical coping strategies to navigate negative affective states.

#### Knowledge of emotional management in illness information

During the transition period, patients expressed diverse needs, especially for informational and emotional support. Many reported limited access to comprehensive information, noting that healthcare professionals often emphasized clinical symptoms while neglecting psychosocial concerns. Participants voiced a strong desire for knowledge on self-management, such as emotional regulation, mental health education, and psychological counseling. Notably, female participants articulated heightened concerns regarding emotional management and self-care during the transition, frequently citing the need for additional support in coping with anxiety and mood fluctuations.

*N1: “Psychotherapists will just give me some reasoning, and I cannot listen to them, I do not think it’s useful and it’s expensive, it’s just a waste of money.”*

*N6: “Difficult to control emotions, easy to cry, regardless of the occasion?”*

*N12: “I found that there is a lot of mixed information about this disease on the internet, some of which are even contradictory, and I do not know which one to trust.”*

#### Coping skills for non-suicidal self-injury

Patients with mental disorders often experienced symptoms like hallucinations and delusions, contributing to self-injurious behaviors. Many reported difficulty managing negative emotions and a strong need for professional support. In cases of non-suicidal self-injury, inadequate emotional regulation, poor coping skills, and limited social support further hinder their ability to cope. These findings underscore the urgent need for targeted psychological interventions and comprehensive support during transitional care.

*N2: “Sometimes suddenly there is a voice in my head that tells me to self-injure, and I do not know how to cope with it.”*

*N7: “I want to know how I should cope with the disease when it strikes, instead of me hiding my feelings.”*

### Contradiction between desire for self-management of illness and limited capacity

Two subordinate themes were identified: (i) a desire to participate in disease management and (ii) over-reliance on parents for illness treatment. Patients often experience a contradiction between their aspiration for autonomy and their limited capacity to manage their condition independently. This theme reflects the psychological complexity and behavioral challenges encountered by adolescents with mental disorders during the transitional period. It underscores the importance of recognizing and balancing patients’ personal desires, functional abilities, and the availability of social support systems. These findings suggest that mental health nursing should adopt a holistic and individualized approach, considering each patient’s readiness and support environment to develop more effective treatment and transition support strategies.

#### Desire to engage in illness management

During adolescence, individuals undergo significant psychological and physiological development with increasing stabilization of personality traits. This developmental stage is also characterized by the emergence of heightened self-awareness and a growing sense of autonomy. Notably, participants in the study expressed a strong desire to actively engage in disease management during the transitional phase. This included assuming responsibility for managing their condition and participating in medical decision-making processes, reflecting their motivation for greater independence and control over their health.

*N2: “When the doctor asks me, my mum always makes decisions for me and answers questions for me, it’s real annoying!”*

*N12: “I know I’m growing up, I wish the doctors and nurses would ask my opinion more often, I should learn to deal with it on my own.”*

#### Psychological dilemma due to limited capacity

In certain cases, parents play a critical role in supporting their children’s daily lives, often assuming responsibility for various aspects of disease management during adolescence. While adolescents may express a desire to independently manage their illnesses, many tasks remain challenging to perform without parental assistance and support. However, excessive reliance on parental involvement may impede the development of adolescents’ self-management skills. Therefore, it is essential to strike an appropriate balance between providing necessary parental support and fostering adolescents’ autonomy to promote their long-term health and self-sufficiency.

*N3: “If I did not have my parents to take care of me, I might have died now. Without them, I could not do many things.”*

*N9: “I cannot do things like going to the hospital and contacting doctors alone, even if I do not want their (parents) help, I still need it in real life.”*

### Dual attributes of family support

Two subordinate themes were identified: (i) the positive motivating effect of family support: family support emerged as a significant protective factor during the transition period. Participants described their families as a source of emotional security, encouragement, and practical assistance. This support bolstered their confidence and sense of stability as they faced new medical and psychological challenges. When encountering uncertainty or distress, the presence of supportive family members helped participants feel less isolated and more empowered to manage their condition. (ii) The obstructive effect of excessive family support: conversely, the analysis also revealed the obstructive potential of excessive family involvement. While families often acted out of concern, their overprotectiveness occasionally hindered the adolescent’s development of autonomy. Some participants reported limited opportunities to manage their own care or make independent decisions, which may impede their ability to build necessary self-management skills.

#### Positive motivating effect of family support

Interviews revealed that adolescents with mental disorders viewed family as a crucial facilitator in the transition to adult healthcare. Family support offered security, reassurance, and emotional strength, boosting their confidence during this critical period. Participants consistently emphasized the pivotal role of family in navigating the challenges of transition, highlighting its essential contribution to a smoother, more supportive process.

*N11: “I used to live on campus, and my mom was worried about what might happen to me. After getting this disease, she rented a house near the school to take care of me, and I also rely more on her parents.”*

*N14: “Their encouragement and help always make me feel at ease, and they will accompany me to see a doctor.”*

#### Obstructive effect of excessive family support

Interviews also revealed that some adolescents viewed their families as obstacles to transition, suggesting that excessive parental control can hinder autonomy and adaptability. This often led to feelings of frustration, confusion, or helplessness. These findings emphasize the need to balance support with independence, highlighting the importance of appropriately calibrated family involvement during the transition to adult mental health services.

*N1: “It’s like I’m being monitored 24 h a day, they think I’m dangerous and suffocating!”*

*N5: “At home, (parents) will urge me to take my medication on time and cook delicious food for me, but sometimes their concern makes me feel like I have not grown up yet and cannot face these difficulties independently.”*

## Discussion

Our study sheds light on the experiences of children and adolescents with mental disorders as they transition to adult healthcare. From an individual perspective, the study found significant differences in patients’ awareness and actions during the transition period. The conflict between the desire to manage the disease and the limited ability to do so highlights the inner struggles faced by patients with multidimensional transition needs. In addition, issues such as incomplete mental healthcare services during the transition period and the interruption of psychological services require attention. It is worth noting that family support has dual attributes. Although family support can be an important facilitator, it can also pose challenges for patients if they are overprotective or exacerbate stress. These themes reveal the challenges and trends faced by adolescent mental health patients in the transition process in the Chinese context and provide valuable insights for clinical practice and policy making.

Our research highlights a significant discrepancy between transitional awareness and actual preparatory actions among children and adolescents with mental disorders. Most participants demonstrated a limited, non-scientific understanding of the transition process and exhibited insufficient motivation to engage in transition-related preparation. Consistent with the findings of Cole (Collet et al., 2025), adolescents with intellectual disabilities and co-occurring mental health disorders face substantial challenges in transitioning to adult care, which could be largely attributed to insufficient preparation and comprehension. Despite these challenges, our study also uncovered a positive trend: participants generally acknowledged the importance of the transition phase for their long-term disease outcomes and expressed a desire to navigate this process effectively. This cognition–action gap calls for targeted interventions to turn their awareness into preparation. From international experience, countries, such as the United States, Europe, and Australia, have accumulated rich experience in transitioning from adolescent to adult care. For example, institutions such as the American Academy of Pediatrics, the American Family Medicine Association, and the American College of Physicians have developed guidelines to facilitate the transition from pediatrics to adult healthcare (Richmond et al., 2012). In China, emerging efforts aim to enhance transitional care for adolescents with chronic illnesses. Gao et al. (2025) developed an information–motivation–Behavior–based transition plan that improved readiness and self-efficacy in adolescents with epilepsy. Studies have also shown that greater willingness to transition is linked to better adaptability, illness management, and treatment adherence (Mlynaryk et al., 2017). Therefore, patients must enhance their transition awareness, understand their condition, acquire health knowledge, maintain a positive outlook, and actively prepare for the transition process.

The findings highlight major challenges in China’s mental healthcare system during the transition from pediatric to adult services, notably fragmented care, poor coordination, and limited service integration. Similar issues have been reported internationally, for instance, Fardell et al. (2017) in Australia revealed significant gaps in the transition from pediatric to adult mental health services, with adolescents often experiencing discontinuity in care due to the lack of integrated service models in in Australia. This study also reveals a systemic neglect of mental health needs during transition, echoing Leeb et al. (2020), who emphasized the need for targeted interventions and heightened awareness at this critical stage. Another key concern is the absence of standardized transition plans, which impedes smooth care continuity. Culnane et al. (2021) similarly found that adolescents often face unstable transitions due to minimal support from general practitioners and a lack of structured preparation. Despite growing awareness, many Chinese institutions still lack formal policies or programs for transitional care. Compared with Western countries, China’s transitional care framework remains underdeveloped and poorly implemented. To address these gaps, interdisciplinary teams, including physicians, nurses, psychotherapists, community providers, and researchers, should be established (Wu and Sun, 2024) to design standardized yet individualized transition plans based on each adolescent’s developmental stage, capabilities, and challenges.

The findings underscore the complex needs of adolescents with mental disorders during the transition to adult healthcare, particularly in emotional regulation and coping with non-suicidal self-injury. A recent survey of mental health professionals in Beijing (Yao et al., 2021) highlighted gaps in family-centered care and limited access to centralized mental health knowledge and transitional skills. Similarly, Fardell et al. (2017) found that adolescents with chronic illnesses often lack transitional skills and informational support due to poor professional guidance and inadequate resource-sharing, issues linked to limited disease-specific knowledge and insufficient training in transitional care. These deficiencies in information and guidance underscore the urgent need for stronger support during this critical transitional period. Prior studies show that effective education on disease management and self-care strongly predicts successful transitions by supporting continuity of care and adaptability (Livanou et al., 2021). To bridge these gaps, healthcare providers should develop targeted education platforms, such as WeChat, phone support, SMS, hospital websites, and social media, to deliver personalized guidance on disease management, psychological support, and skill-building, along with interactive assistance. In addition, this study also found gender differences in transitional experiences: female participants were more concerned with emotional regulation and self-care, reflecting earlier research showing that girls are more likely to seek emotional support and need targeted interventions (Colmenero-Navarrete et al., 2022; Jonsson and Dennhag, 2023). These insights call for gender-sensitive strategies that address specific emotional and psychological needs to improve the effectiveness of transitional care.

The findings highlight a clear tension between adolescents’ desire for autonomy and their limited capacity for self-managing mental health conditions. While many express a strong willingness to participate in their care, they often lack the practical skills and knowledge to do so, leading to continued reliance on parental support. This finding is consistent with the findings of Cui et al. (2023), who found that adolescents with chronic diseases often exhibit a strong desire for independence when managing their condition but often lack the necessary skills, leading to excessive dependence on parental support. This is attributed to parents’ tendency to assume primary responsibility for their child’s daily life and disease management, thereby inadvertently diminishing the adolescent’s self-care and life skills. Notably, previous research (Cui et al., 2023) has established a positive correlation between transitional readiness and self-management efficacy scores among adolescents with chronic diseases. Adolescents who possess a higher level of self-management efficacy tend to have a more favorable attitude toward their illness, utilize available resources more effectively, and engage in better self-management practices. These findings underscore the importance of involving adolescents in making medical decisions based on their age, cognitive level, and emotional maturity. Encouraging such participation fosters autonomy, strengthens self-management, and enhances transition readiness, which is crucial for ensuring continuity of care during the shift to adult mental health services (Huang et al., 2023).

In China, this study provides a new perspective on the dual role of family support during the transition of children and adolescents with mental disorders from pediatric to adult healthcare services. Adequate family support fosters emotional stability, confidence, and readiness for transition. However, excessive parental involvement may hinder the development of self-management and independent living skills, thereby reducing transition readiness. This finding aligns with existing research, emphasizing the need to balance parental involvement and adolescent autonomy for successful healthcare transitions (Xu et al., 2024). In the Chinese sociocultural context, Confucian values, such as filial piety and family harmony, amplify the family’s role in supporting adolescents with mental health conditions (Lam et al., 2022). Filial piety promotes respect and obedience to parents, while parents are expected to offer unconditional care and moral guidance. These values significantly shape mental healthcare in China, where families heavily influence treatment adherence and decision-making (Ma et al., 2023). While culturally rooted and well-intentioned, such involvement can inadvertently undermine adolescents’ self-efficacy and self-regulation. Participants often expressed mixed feelings, grateful for family support but restricted by limited autonomy. This tension underscores the need to calibrate parental involvement to allow adolescents space to develop self-management skills, engage in medical decisions, and gradually take responsibility for their care. Promoting such autonomy is linked to greater self-efficacy, confidence, and improved long-term outcomes. Therefore, a balanced, culturally sensitive approach that combines active family support with strategies to foster adolescent independence is vital for effective transitions.

## Conclusion

This qualitative study deepens our understanding of the transitional experiences and needs of Chinese children and adolescents with mental disorders during the shift from pediatric to adult healthcare services. Key findings include gaps in awareness and action for transition, fragmented transitional care, diverse transitional needs, challenges in self-management, and the dual role of family support. In summary, to better support the transition of Chinese children and adolescents with mental disorders, it is imperative to address their deficiencies in transition preparation and implement corresponding intervention measures. These measures include providing personalized transition support plans, enhancing education and training for patients and families, and fostering effective communication and collaboration between families and healthcare teams. Through these efforts, we can provide more effective nursing support for this particular group, facilitating their smooth transition to adult healthcare and ultimately improving their quality of life and health outcomes.

## Limitations

This study has several limitations that warrant acknowledgment. First, it should be acknowledged that potential interview bias may have arisen due to participants’ age group and clinical setting. The specific characteristics of the participants, including their age and the clinical setting, may have influenced the interview dynamics and responses. Second, it should also be noted that participants were all from a single hospital, which limits generalizability. The findings may not be representative of other hospital settings or broader populations due to the limited sample size and lack of diversity in the study group. Third, limitations of IPA were discussed in interpretive consistency or cultural bias. The use of IPA may introduce variability in how themes are interpreted across different researchers or cultural contexts, potentially affecting the consistency and applicability of the findings.

## Data Availability

The raw data supporting the conclusions of this article will be made available by the authors, without undue reservation.
